# Association of Coffee Consumption With Atrial Fibrillation Risk: An Updated Dose–Response Meta-Analysis of Prospective Studies

**DOI:** 10.3389/fcvm.2022.894664

**Published:** 2022-07-06

**Authors:** Yalin Cao, Xiao Liu, Zhengbiao Xue, Kang Yin, Jianyong Ma, Wengen Zhu, Fuwei Liu, Jun Luo, Junyi Sun

**Affiliations:** ^1^Department of Cardiology, Guizhou Provincial People’s Hospital, Guiyang, China; ^2^Department of Cardiology, Sun Yat-sen Memorial Hospital, Sun Yat-sen University, Guangzhou, China; ^3^Department of Critical Care Medicine, The First Affiliated Hospital of Gannan Medical University, Ganzhou, China; ^4^Department of Pharmacology and Systems Physiology, University of Cincinnati College of Medicine, Cincinnati, OH, United States; ^5^Department of Cardiology, First Affiliated Hospital of Sun Yat-sen University, Guangzhou, China; ^6^Department of Cardiology, The Affiliated Ganzhou Hospital of Nanchang University, Ganzhou, China

**Keywords:** coffee consumption, atrial fibrillation, dose-response, prevention, meta-analysis

## Abstract

**Background:**

Several published studies have examined the association of coffee consumption with atrial fibrillation (AF) risk, but their findings are still controversial. Therefore, we performed a systematic review and dose–response meta-analysis of prospective studies to determine the relationship between coffee consumption and the risk of incident AF.

**Methods:**

We systematically retrieved the PubMed and Embase databases until October 2021 for pertinent studies that reported the association of coffee consumption (caffeinated or decaffeinated coffee) with AF risk. A cubic spline random-effects model was used to fit the potential dose–response curve. The effect estimates were expressed as adjusted risk ratios (RRs) and 95% CIs.

**Results:**

A total of 10 prospective studies (11 cohorts) involving 30,169 AF events and 723,825 participants were included. In the dose–response analysis, there was a linear inverse association between coffee intake and risk of AF although not statistically significant (*P*_non–linearity_ = 0.25). Compared with participants with no coffee consumption, the RRs (95% CI) of AF risk estimated directly from the dose–response curve were 1.01 (0.98–1.03), 1.00 (0.97–1.04), 0.99 (0.92–1.02), 0.95 (0.89–1.01), 0.94 (0.87–1.01), 0.89 (0.79–1.02), and 0.87 (0.76–1.02) for 1–7 cups of coffee per day, respectively. One cup per day increased in coffee consumption was associated with a 2% reduced risk of AF (RR = 0.98, 95% CI: 0.97–1.00, *P* = 0.02).

**Conclusions:**

Our evidence from this meta-analysis suggested that coffee consumption had a trend toward reducing the risk of AF in a dose–response manner. Further studies could be conducted to reinforce our findings.

## Introduction

Atrial fibrillation (AF) is the most common cardiac arrhythmia, with the incident rate increasing consistently over the years (1). The prevalence of AF is estimated to be 12.1 and 17.9 million in the United States and the European Union during 2030–2060, respectively (2). The severe complications of AF such as stroke, heart failure, and sudden death cause an increase in morbidity and mortality (3). Therefore, it is essential to explore the risk factors of AF for prevention. Coffee is one of the most popular beverages globally, while caffeinated products can be found everywhere in life. Recent studies have reported that coffee consumption is positively associated with several acute or chronic diseases (e.g., stroke, Alzheimer’s disease, Parkinson’s disease, type 2 diabetes, and cancer) (4, 5). Also, several studies have shown positive effects of moderate coffee intake on cardiovascular diseases such as heart failure (6) and acute coronary syndrome (7).

However, the dose effects of coffee intake on the risk of AF remain unclear. Due to the excitatory effect of caffeine on sympathetic nerves, coffee or caffeinated products are considered a trigger factor for supraventricular arrhythmias in the 2003 American College of Cardiology/American Heart Association Task Force on Practice Guidelines and the European Society of Cardiology (ACC/AHA/ESC) guidelines for the management of patients with supraventricular arrhythmias (8). Subsequently, ample pieces of evidence have reported an inverse relationship between coffee intake and AF incidents (9–13). As such, the 2020 ESC Guidelines for AF propose that caffeine consumption does not contribute to AF development, and may even reduce the risk of AF (14). Nevertheless, since the sample size and baseline characteristics of participants varied across studies, the data of prior studies lack consensus regarding coffee consumption and AF development. Therefore, we conducted this dose–response meta-analysis of prospective studies to quantitatively determine the relationship between coffee consumption and the risk of incident AF.

## Methods

This meta-analysis was carried out based on the Cochrane Handbook for systemic reviews. The results of this study were reported according to the Preferred Reporting Items for Systematic Reviews and Meta-Analyses (PRISMA) 2020 Statement (15). Ethical approval was not provided because we only included published studies. The data that support the findings of this meta-analysis would be available from the corresponding authors on reasonable request.

### Data Sources and Literature Search

In total, two reviewers (X-L and WG-Z) systematically retrieved relevant studies on the association of coffee consumption with the risk of AF from the PubMed and Embase electronic databases until October 31, 2021. The search terms included two parts: (1) related to the participants of “atrial fibrillation”; and (2) related to the risk factor of “coffee.” These two categories of keywords were combined using the Boolean operator “and.” The search strategies in the electronic databases are shown in Supplementary Table 1. To avoid missing eligible articles, the reference lists of prior meta-analyses and reviews were manually checked for additional studies. This meta-analysis included studies with no language restrictions that met the eligibility criteria.

### Inclusion and Exclusion Criteria

We applied the following inclusion criteria during the study selection. Prospective cohort or nested case–control studies were included if they reported the association of total coffee consumption (caffeinated or decaffeinated coffee) with the risk of AF and/or atrial flutter. There were no strict restrictions on the studied population. Coffee consumption was analyzed as a continuous or categorical variable, and the association with AF risk was expressed as adjusted effect estimates. The assessment methods for AF or atrial flutter were applied according to the originally included studies.

Studies were excluded if they only assessed the relationship between caffeine consumption and AF development. The outcome of postoperative AF was not included. We also excluded certain publication types such as retrospective or cross-sectional studies, case reports, editorials, meeting abstracts, and comments. If facing with overlapping data, we selected the study with the largest sample size or the longest follow-up duration.

### Study Selection and Data Extraction

The PRISMA flow diagram was applied to guide the process of study selection. Studies were selected by two reviewers independently based on the eligibility criteria mentioned above. We first screened the titles and abstracts for the potential studies, and then selected the final included studies after the full-text screenings. Disagreements were resolved through discussion or consultation with a third researcher.

Data from each included study were extracted by two reviewers independently, which were further confirmed by a third researcher to ensure accuracy. We extracted information including the study characteristics (first author, year of publication, location, data source, study design), study participants (age, gender, total sample size, and the number of events), coffee information (caffeinated or decaffeinated coffee, categorization of coffee consumption), ascertainment of AF, adjusted factors in the multivariable analysis, follow-up period, and outcome data (sample size and the number of events in the groups, adjusted effect estimates). If the included studies reported adjusted effect estimates in multiple models or different follow-up periods, we applied the most adjusted one or the effect estimate in the longest follow-up period.

### Quality Assessment

The quality assessment was performed using the Newcastle-Ottawa Scale (NOS) tool. This tool had three domains ranging from 0 to 9 points, including the selection of cohorts (0–4 points), the comparability of cohorts (0–2 points), and the assessment of the outcome (0–3 points). According to the previous studies (16, 17), the NOS of ≥ 6 and < 6 points were considered moderate-to-high quality and low-quality, respectively.

### Consistency Test and Publication Bias

The statistical heterogeneity across the included studies was assessed using the *P*-value of the Cochrane *Q* test and the *I*^2^ value, where *P* < 0.1 or *I*^2^ > 50% suggested significant between-study heterogeneity. If facing an *I*^2^ value of >50%, we excluded each included study at a time to find out the potential source of high heterogeneity. We assessed the potential risk of publication bias by visually inspecting the funnel plots. In addition, the statistical publication bias was further assessed using the Egger’s and Begg’s tests, where a *P*-value of >0.1 indicated no significant publication bias.

### Statistical Analysis

Both semiparametric and parametric methods were applied to assess the effect of coffee consumption on the risk of AF. In the semiparametric methods, the categorizations of coffee consumption included three parts, namely, lowest, second highest, and highest coffee intake levels according to the reports on coffee consumption and risk of diabetes (18). The association with AF risk was expressed as adjusted risk ratios (RRs) and 95% confidence intervals (CIs). A DerSimonian and Laird random-effects model with an inverse variance method was applied to pool the RRs. For the parametric method, dose-response associations (linear and non-linear) of coffee consumption and risk of AF were assessed. In all included studies, coffee intake (e.g., cups/week) was converted into a uniform unit (cups/day). Study-specific risk estimates (per 1 cup/day coffee intake) and 95% CIs from the natural logs of the reported risk estimates and CIs across categories were calculated using Greenland and Longnecker (19). For non-linear analysis, a one-stage robust error meta-regression method was used (20). It required at least two categories of coffee intake and the corresponding risk estimates with variance estimates. For these studies with no median or mean coffee intake level in each category, the midpoint of each category was estimated (21, 22). If the highest or lowest category was open-ended, the open-ended interval length was assumed to be that of the adjacent interval (21, 22).

In the sensitivity analysis, re-analysis with a fixed-effects model was applied. In addition, we also re-performed the analysis after excluding the studies with data on decaffeinated coffee, or the healthy population. In addition, the included studies with adjustment for age, sex, ethnicity, education, smoking, alcohol intake, body mass index, physical activity, diabetes, hypertension, coronary heart disease, or heart failure were separately pooled. The subgroup analysis was performed based on sex (males vs. females).

All the statistical analyses were performed using the Review Manager version 5.4 software (the Cochrane Collaboration 2014, Nordic Cochrane Centre Copenhagen, Denmark^1^), and the Stata software (version 15.0, StataCorp LP, College Station, TX, United States). In this study, a *P* < 0.05 indicated statistical significance.

## Results

### Study Identification and Selection

**FIGURE 1:**
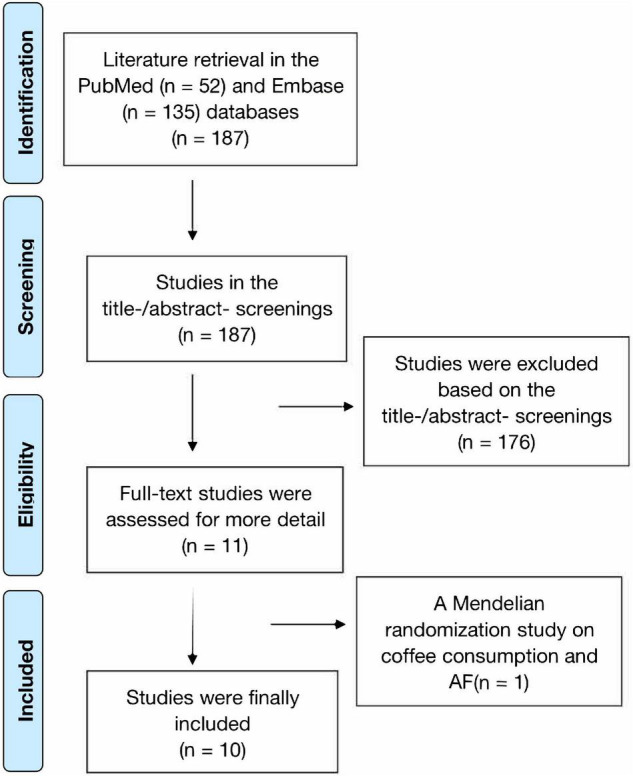
Document retrieval flow chart of this meta-analysis.

The process of literature retrieval is shown in Figure 1. A total of 187 records were retrieved from the PubMed (*n* = 52) and Embase (*n* = 135) databases. We found no additional studies through the reference lists of prior meta-analyses and reviews (23–25). After the first (titles and abstracts) and second (full text) phases of screening, 11 remaining studies were potentially available. In addition, a Mendelian randomization study on coffee consumption and AF risk was further excluded due to insufficient data (26). Finally, a total of 10 prospective studies (9–13, 27–31) were included in this meta-analysis. Since Bazal et al. (9) reported two cohorts of Seguimiento Universidad de Navarra (SUN) and Prevencion con Dieta Mediterranea (PREDIMED), we included 11 cohorts in the current study (9–13, 27–31).

### Study Characteristics

Table 1 shows the baseline characteristics of the included studies. Data on caffeinated coffee were reported in three studies (9, 29, 30), and only one study by Conen et al. (29) presented the data on decaffeinated coffee. Results of caffeinated and decaffeinated coffee were mixed in the remaining studies. Categorizations of coffee intake differed among all the studies, and coffee consumption was analyzed as a continuous variable in two studies (11, 12). The follow-up time across studies ranged from 4.4 to 25.2 years. Ascertainment of AF in each study was mainly conducted through electrocardiography, codes of the international classification of diseases, or medical records. Klatsky et al. (12) reported the outcome of AF and atrial flutter separably, and thus, we combined them in the pooled analysis.

**TABLE 1:** Baseline characteristics of the included studies of this meta-analysis.

Studies (references)	Data source and study design	Population	Age (years)	Sex	AF events	Sample size	Categorization of coffee consumption	Ascertainment of AF	Adjustments	Follow-up (years)
(31)	Multifactor primary Prevention study; Sweden	General population	47–55	100% males	754	7495	Undefined coffee^#^: 0; 1–4; ≥ 5 cups/day	ICD codes	Age	25.2
(30)	Stockholm Heart epidemiology program; Sweden	Patients who had survived a MI and free from AF	47–50	Both	163	1369	Caffeinated coffee: 0–1; 1–3; 3–5; 5–7; ≥ 7 cups/day	ICD codes	Age, sex, education, smoking, diabetes, obesity, physical inactivity, alcohol, tea and boiled coffee	6.9–9.9
(29)	Women’s health study; United States	Healthy population without cardiovascular disease and AF	≥ 45	100% females	936	NA	Caffeinated coffee*: 0; 0–1; 1; 2–3; ≥ 4 cups/day	Self reported; medical records	Age, race/ethnicity, treatment group, SBP, hypertension, hypercholesterolemia, smoking, diabetes, BMI, exercise, parental history of MI, intake of alcohol and fish	14.4
(12)	California comprehensive health care plan; United States	General population	NA	Both	1785	130054	Caffeinated and decaffeinated coffee: 0; 0–1; 1–3; ≥ 4 cups/day; Per cup per day	ICD codes	Age, sex, ethnicity, BMI, education, cigarette smoking, a cardiorespiratory composite covariate, alcohol intake	17.6
(28)[men]	Cohort of Swedish men	General population free from AF	45–79	100% males	4311	41881	Undefined coffee^#^0–2; 2–3; 3–4; 4–5; ≥ 5 cups/day	ICD codes	Age, education, smoking, histories of cardiac disease, hypertension, diabetes, BMI, PA, family history of MI, intake of alcohol and tea	12.0
(28)[women]	Swedish Mammography Cohort	General population free from AF	49–83	100% females	2730	34594	Undefined coffee^#^0–2; 2–3; 3–4; 4–5; ≥ 5 cups/day	ICD codes	Age, education, smoking, histories of cardiac disease, hypertension, diabetes, BMI, PA, family history of MI, and intake of alcohol and tea	12.0
(13)	Danish diet, cancer, and health study	General population free from AF	50–64	Both	3415	57053	Caffeinated and decaffeinated coffee: 0; 0–1; 1; 2–3; 4–5; 6–7; ≥ 7 cups/day	ICD codes	Age, sex, BMI, SBP, total serum cholesterol, alcohol, smoking, education, hypertension, diabetes, cardiovascular disease	13.5
(10)	Physicians’ health study; United States	Male physicians free from AF	Mean 66.1	100% males	2098	18960	Caffeinated and decaffeinated coffee: 0; 1; 2–3; ≥ 4 cups/day	Self reported; medical records	Age, smoking, alcohol, exercise, BMI, SBP, taking antihypertensives, diabetes, high cholesterol, HF, CHD	9.0
(27)	Multi-ethnic study of atherosclerosis; United States	Healthy population without cardiovascular disease and AF	45–84	Both	828	5972	Caffeinated and decaffeinated coffee: 0; 0–0.5; 0.5–1.5; ≥ 1.5 cups/day	ECG; ICD codes	Age, sex, race/ethnicity, education, BMI, SBP, DBP, taking antihypertensives, diabetes, LDL, HDL, alcohol, smoking, daily soda intake, daily diet soda intake, daily tea intake	14.0
(9)	SUN cohort; Spain	General population free from AF	Mean 37.5	Both	97	18983	Caffeinated coffee*: < 3 cups/month; 1–7 cups/week; > 1 cup/day	Self reported; ECG; medical records	Age, sex, smoking, BMI, height, PA, sleep apnoea, diabetes, hypertension, alcohol, ischemic cardiopathy, HF, other caffeinated beverages and decaffeinated coffee	10.3
(9)	PREDIMED cohort; Spain	Elderly free from cardiovascular disease but at high cardiovascular risk	55–80	Both	250	6479	Caffeinated coffee*: < 3 cups/month; 1–7 cups/week; >1 cup/day	ECG	Age, sex, smoking, BMI, height, PA, sleep apnoea, diabetes, SBP, DBP, hypertension, alcohol, HF, other caffeinated beverage, decaffeinated coffee, intervention group, depression	4.4
(11)	UK biobank	General population free from tachyarrhythmias	40–69	Both	12811	386258	Caffeinated and decaffeinated coffee: Per cup per day	ICD codes	Age, sex, ethnicity, BMI, education, hypertension, diabetes, hyperlipidemia, CHD, HF, valvular heart disease, cerebrovascular disease, peripheral artery disease, chronic kidney disease, cancer, smoking, alcohol, tea consumption, PA	4.5

*^#^Caffeinated and decaffeinated coffee were not stated in the original study.*

*Population taking caffeinated coffee was used for analysis, some of who could also be on decaffeinated coffee.

*AF, atrial fibrillation; BMI, body mass index; MI, myocardial infarction; PA, physical activity; CHD, coronary heart disease; SBP, systolic blood pressure; DBP, diastolic blood pressure; HF, heart failure; LDL, low density lipoprotein; HDL, high density lipoprotein; SUN, Seguimiento Universidad de Navarra; PREDIMED, Prevencion con Dieta Mediterranea; ECG, electrocardiography; ICD, International Classification of Diseases; NA, not available.*

### Risk of Bias Within Studies

The representativeness of the three exposed cohorts was relatively limited. Mukamal et al. (30) included patients who had survived a myocardial infarction, Bodar et al. (10) enrolled male physicians without AF at baseline, whereas Bazal et al. (9) included the elderly free from cardiovascular disease but at high cardiovascular risk in the PREDIMED cohort. Ascertainment of coffee consumption in the study of Wilhelmsen et al. (31) was not defined. Whether the baseline AF was excluded was not reported in two studies (12, 31). The length of follow-up was less than 5 years in two studies (9, 11). Overall, all included studies had moderate-to-high quality with a NOS of ≥ 6 points (Supplementary Table 2).

## Synthesis of Results

### Categorical Analysis of Total Coffee Consumption and Atrial Fibrillation Risk

**FIGURE 2:**
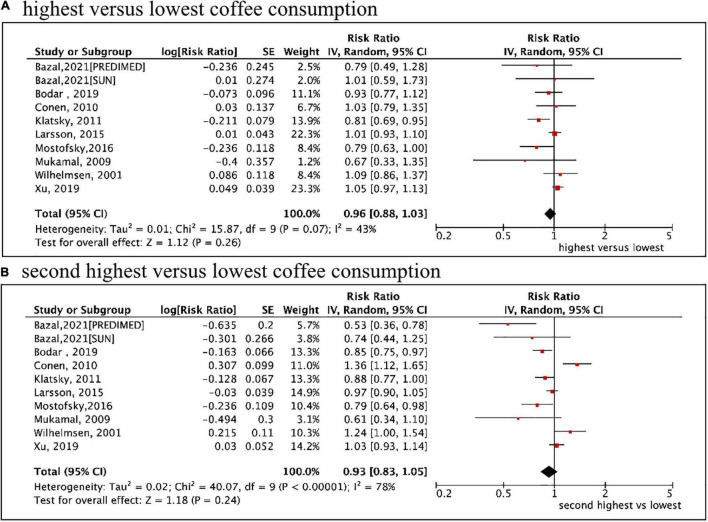
Categorical analysis of the associations of total coffee consumption with the risk of atrial fibrillation. **(A)** The highest (median ≥ 4 cups/d) vs. lowest coffee intake level. **(B)** The second highest (median 2.5 cups/d) vs. lowest coffee intake level.

A total of 9 included studies (10 cohorts) (9–13, 27–31) with 10,670 cases/337,567 participants were included for analysis regarding the association of coffee intake (as a category variable) on AF risk. In the primary analysis from a random-effects model, the pooled results showed that neither the highest (median ≥ 4 cups/d) vs. the lowest coffee intake level (RR = 0.96, 95% CI 0.88–1.03, *I*^2^ = 43%) nor the second-highest (median 2.5 cups/d) vs. the lowest coffee intake level (RR = 0.93, 95% CI 0.88–1.03, *I*^2^ = 78%) was significantly associated with an increased risk of AF (Figure 2). We failed to find the potential source of heterogeneity after excluding studies one by one. Nevertheless, the corresponding results were stable after omitting each included study at a time.

The sensitivity analysis obtained similar results using a fixed-effects model (Supplementary Figure 1). As shown in Table 2, the pooled data of 8 cohorts enrolling the healthy population or 4 cohorts reporting caffeinated coffee suggested no significant associations of coffee consumption with AF risk. After adjusting for age, sex, ethnicity, education, smoking, alcohol intake, body mass index, physical activity, diabetes, hypertension, or coronary heart disease, the pooled results were stable with consistent findings with the primary analysis. The second highest vs. the lowest level of coffee intake was significantly associated with a reduced risk of AF (RR = 0.72, 95% CI 0.53–0.98) after adjusting for heart failure.

**TABLE 2:** Pooled effect estimates for coffee consumption and AF risk.

	Highest (median ≥ 4 cups/d) vs. lowest				Second highest (median 2.5 cups/d) vs. lowest			Per cup/Day			
			
	No. of effect estimates	RRs and 95% CIs	P value	I^2^ statistic	RRs and 95% CIs	P value	I^2^ statistic	No. of effect estimates	RRs and 95% CIs	P value	I^2^ statistic
**Overall**											
*Random model*	10	0.96 [0.88, 1.03]	0.26	43%	0.93 [0.83, 1.05]	0.24	78%	10	0.98 [0.97, 1.00]	0.02	61%
*Fixed model*	10	0.99 [0.94, 1.04]	0.62	43%	0.96 [0.92, 1.01]	0.09	78%	10	0.98 [0.97, 0.99]	< 0.00001	61%
**Subgroup analysis**											
*Men*	3	1.05 [0.97, 1.14]	0.26	0%	0.99 [0.83, 1.17]	0.88	77%	3	1.00 [0.97, 1.02]	0.81	52%
*Women*	2	0.91 [0.80, 1.04]	0.18	2%	1.14 [0.83, 1.58]	0.41	87%	−	−	−	−
**Sensitivity analysis**											
*Caffeinated coffee*	4	0.94 [0.77, 1.16]	0.58	0%	0.77 [0.45, 1.34]	0.36	87%	3	0.92 [0.78, 1.08]	0.30	27%
*Healthy population*	8	0.96 [0.89, 1.05]	0.39	49%	0.98 [0.88, 1.09]	0.71	76%	8	0.98 [0.97, 1.00]	0.04	64%
*Adjusted for age*	10	0.96 [0.88, 1.03]	0.26	43%	0.93 [0.83, 1.05]	0.24	78%	10	0.98 [0.97, 1.00]	0.02	61%
*Adjusted for sex*	9	0.94 [0.87, 1.03]	0.17	47%	0.90 [0.80, 1.02]	0.10	77%	9	0.98 [0.96, 1.00]	0.02	64%
*Adjusted for ethnicity*	3	0.95 [0.79, 1.13]	0.54	77%	1.06 [0.86, 1.30]	0.58	85%	2	0.92 [0.74, 1.13]	0.41	86%
*Adjusted for education*	5	0.93 [0.82, 1.05]	0.22	71%	0.93 [0.84, 1.02]	0.12	56%	5	0.99 [0.96, 1.01]	0.23	0%
*Adjusted for smoking*	9	0.94 [0.87, 1.03]	0.17	47%	0.90 [0.80, 1.02]	0.10	77%	9	0.98 [0.96, 1.00]	0.02	64%
*Adjusted for alcohol*	9	0.94 [0.87, 1.03]	0.17	47%	0.90 [0.80, 1.02]	0.10	77%	9	0.98 [0.96, 1.00]	0.02	64%
*Adjusted for* body mass index	8	0.95 [0.87, 1.03]	0.22	50%	0.92 [0.81, 1.03]	0.16	78%	8	0.98 [0.96, 1.00]	0.04	68%
*Adjusted for physical activity*	5	0.93 [0.81, 1.06]	0.27	0%	0.81 [0.58, 1.13]	0.22	85%	5	0.97 [0.96, 0.98]	< 0.00001	0%
*Adjusted for diabetes*	8	0.99 [0.93, 1.06]	0.79	17%	0.90 [0.79, 1.04]	0.15	79%	8	0.98 [0.97, 1.00]	0.02	58%
*Adjusted for hypertension*	7	0.99 [0.93, 1.06]	0.85	16%	0.92 [0.80, 1.06]	0.24	80%	7	0.98 [0.97, 1.00]	0.04	62%
*Adjusted for* coronary heart disease	3	0.97 [0.83, 1.12]	0.64	0%	0.98 [0.67, 1.43]	0.92	88%	2	0.97 [0.94, 1.01]	0.16	0%
*Adjusted for* heart failure	3	0.92 [0.78, 1.09]	0.32	0%	0.72 [0.53, 0.98]	0.04	61%	3	0.93 [0.78, 1.11]	0.42	35%

*AF, atrial fibrillation; RR, risk ratio; CI, confidence interval.*

### Dose–Response Association of Total Coffee Consumption With Atrial Fibrillation Risk

**FIGURE 3:**
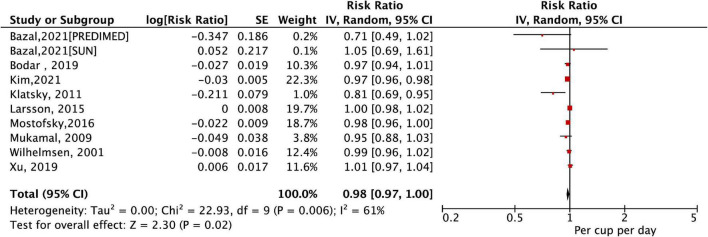
The association of a per cup/day increase in coffee consumption with the risk of atrial fibrillation.

A total of 10 studies (11 cohorts) (9–13, 27–31) with a total of 30,169 cases/723,825 participants were included in the dose–response analysis. As shown in Figure 3, the pooled results by a random-effects model showed that a per cup/day increase in coffee consumption was significant with a decreased risk of AF (RR = 0.98, 95% CI: 0.97–1.00, *P* = 0.02). In the sensitivity analysis, a fixed-effects model analysis generated similar results (Supplementary Figure 2). Per cup/day of coffee, intake was significantly associated with a decreased risk of AF in the healthy population. This reduced association was also observed after adjusting for age, sex, smoking, alcohol intake, body mass index, physical activity, or diabetes (Table 2).

**FIGURE 4:**
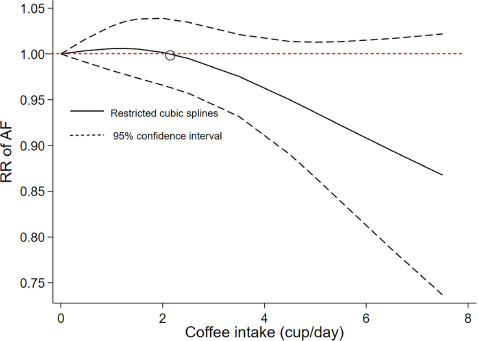
Dose–response association of coffee intake with the risk of atrial fibrillation assessed by a cubic spline model.

As shown in Figure 4, a cubic spline model was used to fit the potential dose–response curve. The results showed a linear inverse association between coffee intake and the risk of AF although not statistically significant (*P*_non–linearity_ = 0.25). Compared with participants with no coffee consumption, the risk of AF estimated directly from the dose–response curve for 1–7 cups/day were 1.01 (95% CI 0.98–1.03), 1.00 (0.97–1.04), 0.99 (0.92–1.02), 0.95 (0.89–1.01), 0.94 (0.87–1.01), 0.89 (0.79–1.02), and 0.87 (0.76–1.02), respectively.

### Subgroup Analysis

In the categorical analysis of the association of coffee intake with AF risk, there were no interactions between sex regarding the association of the highest (*P*_interaction_ = 0.08; Supplementary Figure 3) or second-highest (*P*_interaction_ = 0.42; Supplementary Figure 4) vs. lowest category of coffee intake with AF risk. In addition, there was no difference in the sex-based analysis for the association of per cup/day of coffee intake with AF risk (*P*_interaction_ = 0.48; Supplementary Figure 5).

### Publication Bias

As shown in Supplementary Figure 6, the funnel plots suggested no potential publication bias. The results from the Egger’s and Begg’s tests also showed no significant publication bias (all *P* > 0.1; Supplementary Figure 7).

## Discussion

To our knowledge, this was the first meta-analysis including 10 studies with a total of 723,825 participants to assess the association of coffee consumption with the risk of incident AF. Our pooled results suggested that (1) neither the highest (median ≥ 4 cups/d) nor the second-highest (median 2.5 cups/d) coffee intake level compared with the lowest level was associated with an increased risk of AF; (2) one cup per day increase in coffee consumption was with a decreased risk of AF. There was a linear inverse relationship between coffee consumption and AF risk although not significant, suggesting that AF risk tended to decrease with higher intakes. Overall, our current evidence indicated that moderate to high levels of coffee consumption at least did not increase the incidence of AF.

A prior meta-analysis conducted by Krittanawong et al. (23) included 7 prospective and 5 retrospective cohorts with a total of 361,143 individuals to examine the association between caffeine or coffee consumption and AF risk, suggesting that a mixed analysis of caffeine or coffee products did not increase the risk of AF. Caldeira et al. (32) acquired similar findings by analyzing a total of 115,993 participants from 7 observational studies on caffeine exposure and AF risk. However, these two pooled analyses mixed coffee and caffeine consumption. The comparison between our study and previous meta-analyses was summarized in Supplementary Table 3. The result of our study that coffee was not a trigger of AF was kept consistent with most of the previous ones, while we analyzed the dose-response manner additionally and showed a potential protective effect with more cups of coffee intake. Although the number of studies involved in this meta-analysis was relatively small, all included were high-quality prospective studies with large sample sizes, large numbers of cases, pure coffee, and long follow-up times, making the results of this study more reliable. Previously, Mostofsky et al. found a J-shaped relationship to heart failure, with the maximum reduction effect occurring at the three cups of coffee a day (6). Another study conducted by Zhang et al. presented that 1–3 cups/day of coffee might increase the risk of hypertension, whereas more than three cups/day had no effects anymore (33). Our present study also found a tendency that 1–2 cups of coffee a day might slightly increase the risk of AF, whereas intake of more than two cups/day may reduce the onset of AF. The risk of AF tended to decrease with higher intakes of coffee consumption.

As a complex beverage roasted from plants, coffee contains more than 1,000 biologically active chemicals, including caffeine, chlorogenic acids, diterpenes, cafestol, kahweol, trigonelline, melanoidins, and modest amounts of electrolytes and vitamins (34). Some of these chemicals such as diterpenes cafestol and kahweol can significantly raise the serum levels of total and low-density lipoprotein cholesterol, whereas other components of coffee are the potential protective factors due to their antioxidant, anti-platelet, and anti-inflammatory properties (11, 34, 35). The cumulative effects of coffee consumption are potentially attributed to a combination of positive and negative actions of different coffee components (34). Several mechanisms of coffee-related arrhythmias have been revealed over the years. Caffeine, the most studied ingredient in coffee, is widely thought to play an important role in a neuroendocrine pathway that increases serum renin, norepinephrine, and epinephrine concentrations (36) and delays ventricular myocyte afterdepolarizations by releasing calcium from the sarcoplasmic reticulum (37). Adenosine is a small molecule mainly generated by ATP catabolism and will increase during the pathogenic processes of inflammation, hypoxia, and ischemia (38). Excessive release of adenosine or overexpression of adenosine receptors in the heart causes atrial electrophysiological abnormalities, eventually causing AF (38). Caffeine can delay this process as the blocker of adenosine receptors (39). In addition, the anti-inflammatory and antioxidant effects of coffee may prevent the occurrence of AF (34, 40). To strengthen the hypothesis, Lara-Guzman et al. (35) conducted a randomized controlled trial revealed that 8-week coffee consumption could decrease oxidized and inflammatory components (e.g., oxysterol, free fatty acid) in healthy subjects by analyzing lipidomic data of 46 lipids, while the changes were not associated with the concentration of chlorogenic acids, an important antioxidant in coffee.

In our pooled analysis, we combined caffeine and decaffeinated coffee as total coffee due to the lack of an exact number of participants consuming either kind of coffee in every included study. Additional data from four cohort studies reporting caffeinated coffee still suggested no positive associations between coffee consumption with AF risk. Decaffeinated coffee was not analyzed in our study due to limiting data. However, different brewing methods would make the coffee components differ a lot (4, 34), which might explain the inconsistent results of studies conducted in different regions of the world. Conen et al. (29) found that for healthy middle-aged women, consumption of 2–3 cups of caffeinated coffee per day increased the risk of incident AF compared with coffee-naïve people, whereas the identical cups of decaffeinated coffee did not influence the AF risk, suggesting a potential bias due to the neutral effects of decaffeinated coffee that weaken the actual impact of caffeinated coffee on AF risk. Meanwhile, we could not exclude the influence of other components of coffee on the onset of AF due to the constitution and concentration varying among different brewing methods. Caffeine can be found in many beverages or foods, ranging from 40 to 180 mg in coffee, 24–50 mg in tea, 15–29 mg in cola, and 1–36 mg in chocolate. When taking caffeine-contained beverages and food into consideration, Mattioli et al. (41) showed that dietary sources of caffeine would reduce AF episodes. Therefore, the results of this study may be partly related to other sources of caffeine.

Recently, Marcus et al. designed an n-of-1 randomized clinical trial and found that except for alcohol, other self-selected triggers (coffee, exercise, and dehydration) are neither related to the AF-related quality of life nor the onset of AF (42). The n-of-1 trial used in this study was highly personalized and could help select the most effective one from various treatments in a relatively short time so that patients could directly benefit from it. Our current data from this meta-analysis found that a per cup/day increase in coffee consumption had a potential preventive effect, which might guide the dietary habits to prevent AF. Further large-scale randomized clinical trials are still needed to obtain more universal conclusions about the association of coffee consumption with AF development.

### Limitations

Our analysis had several limitations that should be carefully addressed. First, because caffeinated and decaffeinated coffee may have different effects on AF risk, future studies should examine subgroups based on different types of coffee separately. Second, in the studies we included, coffee consumption was calculated based on individual reports, and they did not explicitly state the brewing methods of coffee. Therefore, the exact volume could not be accurately obtained, nor did the content of various ingredients in the coffee. Third, the studies included in this meta-analysis were mainly conducted in the United States and European countries. Future research in different populations would help draw conclusions about the relationship between coffee consumption and the risk of AF worldwide. Finally, no strict restrictions on the populations were applied during the inclusions of studies. Nevertheless, we separately pooled 8 cohorts enrolling the healthy population, suggesting similar findings.

## Conclusion

Our meta-analysis suggested that coffee consumption had a trend in reducing the risk of AF in a dose-response manner. Coffee intake at least did not increase the incidence of AF, conveying to public health that coffee intake was not a trigger of AF development.

## Data Availability Statement

The original contributions presented in this study are included in the article/Supplementary Material, further inquiries can be directed to the corresponding authors.

## Author Contributions

All authors listed have made a substantial, direct, and intellectual contribution to the work, and approved it for publication.

## Conflict of Interest

The authors declare that the research was conducted in the absence of any commercial or financial relationships that could be construed as a potential conflict of interest.

## Publisher’s Note

All claims expressed in this article are solely those of the authors and do not necessarily represent those of their affiliated organizations, or those of the publisher, the editors and the reviewers. Any product that may be evaluated in this article, or claim that may be made by its manufacturer, is not guaranteed or endorsed by the publisher.

## Footnotes

1
https://community.cochrane.org/
