# Supplementary material for: Association of Coffee Consumption With Atrial Fibrillation Risk: An Updated Dose–Response Meta-Analysis of Prospective Studies
Source: Front Cardiovasc Med. 2022 Jul 6;9:894664. doi: 10.3389/fcvm.2022.894664 (PMC9299433; doi:10.3389/fcvm.2022.894664)
Supplement: Supplementary file 1 [file Data_Sheet_1.PDF]

Supplemental Table 1. The search strategies of this meta-analysis until October, 2021

	Search terms	Items
**PubMed**		
#1	atrial fibrillation	93881
#2	atrial flutter	10011
#3	coffee	17660
#4	((atrial flutter) OR (atrial fibrillation)) AND (coffee)	52
**Embase**		
#1	atrial fibrillation	193774
#2	atrial flutter	17234
#3	coffee	24262
#4	((atrial flutter) OR (atrial fibrillation)) AND (coffee)	135

Supplemental Table 2. Quality assessment of the included studies based on the NOS tool

**Studies**	**Selection**				**Comparability**	**Outcome**			**Total**
	**Exposed cohort**	**Non-exposed cohort**	**Ascertainment of exposure**	**Outcome of interest**		**Assessment of outcome**	**Length of follow-up**	**Adequacy of follow up**	
Wilhelmsen, 2001	*	*			*	*	*	*	6
Mukamal, 2009		*	*	*	**	*	*	*	8
Conen, 2010	*	*	*	*	**	*	*	*	9
Klatsky, 2011	*	*	*		**	*	*		7
Larsson, 2015	*	*	*	*	**	*	*	*	9
Mostofsky,2016	*	*	*	*	**	*	*	*	9
Xu, 2019	*	*	*	*	**	*	*	*	9
Bodar， 2019		*	*	*	**	*	*	*	8
Bazal,2021a	*	*	*	*	**	*		*	7
Bazal,2021b		*	*	*	**	*		*	8
Kim, 2021	*	*	*	*	**	*		*	8

NOS=Newcastle-Ottawa Scale.

Supplemental Table3. Comparison between previous meta-analyses and current study

Studies	Included studies	Total participants	Exposure	Conclusion
Caldeira, 2013	1 case-control study<br>6 observational cohorts<br>(5 prospective, 1 unclear)	115,993	caffeine	Caffeine exposure is not associated with increased AF risk.<br>Low-dose caffeine may have a protective effect.
Cheng, 2014	6 prospective cohorts	228,465	caffeine	It is unlikely that caffeine consumption causes or contributes to AF.<br>Habitual caffeine consumption might reduce AF risk.
Larsson, 2015	6 prospective cohorts	248,910	coffee	Coffee consumption is not associated with increased risk of AF.
Abdelfattah, 2018	1 case-control study<br>7 observational cohorts<br>(6 prospective, 1 unclear)	176,675	caffeine	The incidence of AF is not increased by coffee consumption.<br>lower incidence of AF when caffeine consumption exceeded 436 mg/day.
Krittanawong, 2021	7 prospective cohorts<br>5 retrospective cohorts	361,143	caffeine or coffee	caffeine or coffee consumption is not associated with the risk of new-onset AF.
Current study	10 prospective cohorts	723,825	coffee	Coffee consumption had a trend in reducing the risk of AF in a dose-response manner.<br>Coffee intake at least did not increase the incidence of AF.

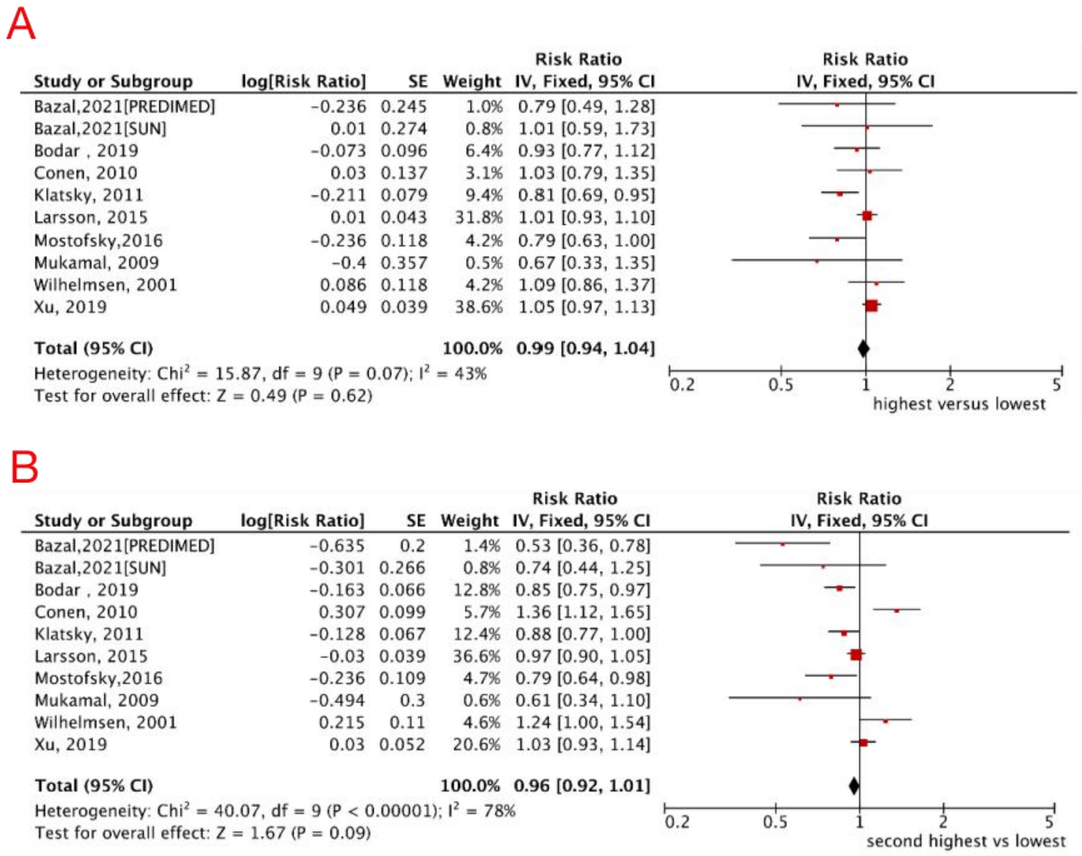

Supplemental Figure 1. Categorical analysis of the associations of coffee intake with AF risk assessed by a fixed-effects model (A:highest versus the lowest coffee intake level; B:second-highest versus the lowest coffee intake level)

Study or Subgroup	log[Risk Ratio]	SE	Weight	Risk Ratio IV, Fixed, 95% CI
Bazal,2021[PREDIMED]	−0.347	0.186	0.0%	0.71 [0.49, 1.02]
Bazal,2021[SUN]	0.052	0.217	0.0%	1.05 [0.69, 1.61]
Bodar , 2019	−0.027	0.019	3.5%	0.97 [0.94, 1.01]
Kim,2021	−0.03	0.005	50.6%	0.97 [0.96, 0.98]
Klatsky, 2011	−0.211	0.079	0.2%	0.81 [0.69, 0.95]
Larsson, 2015	0	0.008	19.8%	1.00 [0.98, 1.02]
Mostofsky,2016	−0.022	0.009	15.6%	0.98 [0.96, 1.00]
Mukamal, 2009	−0.049	0.038	0.9%	0.95 [0.88, 1.03]
Wilhelmsen, 2001	−0.008	0.016	4.9%	0.99 [0.96, 1.02]
Xu, 2019	0.006	0.017	4.4%	1.01 [0.97, 1.04]
**Total (95% CI)**			**100.0%**	**0.98 [0.97, 0.99]**

Heterogeneity: $Chi^2 = 22.93$, $df = 9$ ($P = 0.006$); $I^2 = 61\%$

Test for overall effect: $Z = 5.81$ ($P < 0.00001$)

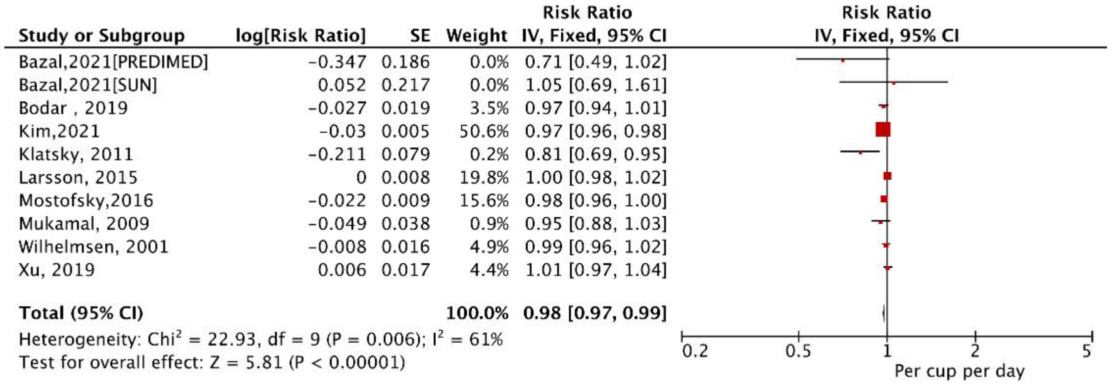

Supplemental Figure 2. Association of per cup/day of coffee intake with AF risk assessed by a fixed-effects model

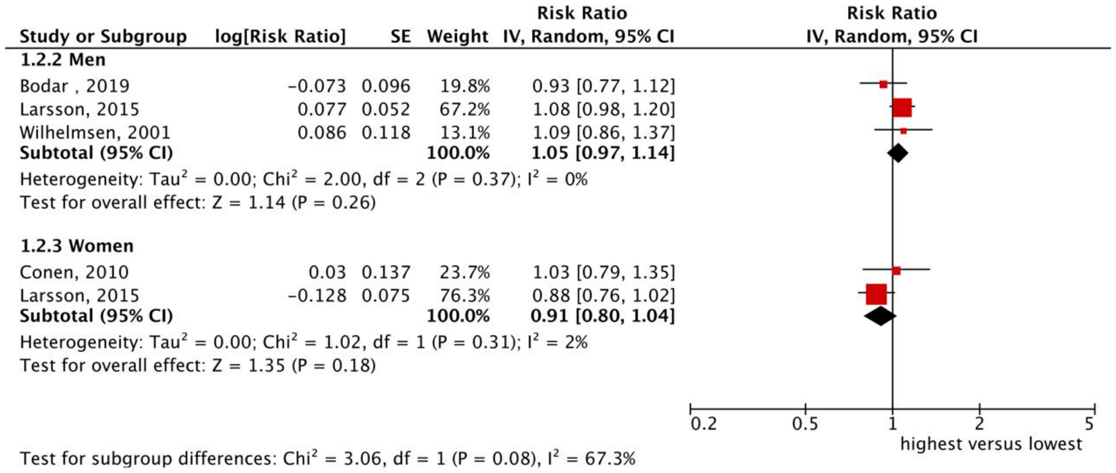

Supplemental Figure 3. Sex-based analysis for AF risk between the highest versus lowest category of coffee consumption

Study or Subgroup	log[Risk Ratio]	SE	Weight	Risk Ratio IV, Random, 95% CI
**1.2.2 Men**				
Bodar , 2019	−0.163	0.066	35.5%	0.85 [0.75, 0.97]
Larsson, 2015	−0.03	0.053	38.2%	0.97 [0.87, 1.08]
Wilhelmsen, 2001	0.215	0.11	26.3%	1.24 [1.00, 1.54]
**Subtotal (95% CI)**			**100.0%**	**0.99 [0.83, 1.17]**
**1.2.3 Women**				
Conen, 2010	0.307	0.099	47.5%	1.36 [1.12, 1.65]
Larsson, 2015	−0.02	0.067	52.5%	0.98 [0.86, 1.12]
**Subtotal (95% CI)**			**100.0%**	**1.14 [0.83, 1.58]**

Men — Heterogeneity: $Tau^2 = 0.02$; $Chi^2 = 8.86$, df = 2 (P = 0.01); $I^2 = 77\%$
Test for overall effect: Z = 0.15 (P = 0.88)

Women — Heterogeneity: $Tau^2 = 0.05$; $Chi^2 = 7.48$, df = 1 (P = 0.006); $I^2 = 87\%$
Test for overall effect: Z = 0.83 (P = 0.41)

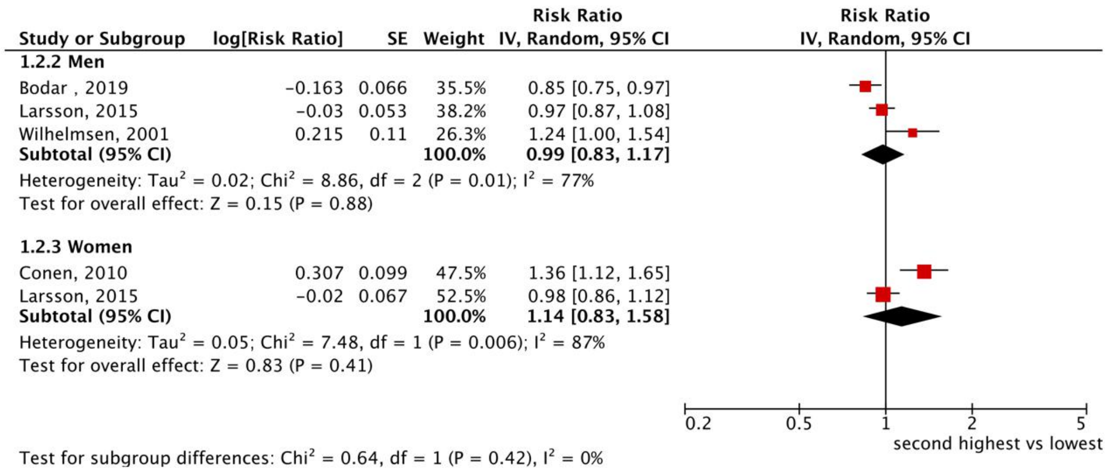

Test for subgroup differences: $Chi^2 = 0.64$, df = 1 (P = 0.42), $I^2 = 0\%$

Supplemental Figure 4. Sex-based analysis for AF risk between the second-highest versus lowest category of coffee

Study or Subgroup	log[Risk Ratio]	SE	Weight	Risk Ratio IV, Random, 95% CI
**1.2.2 Men**				
Bodar , 2019	−0.027	0.019	25.0%	0.97 [0.94, 1.01]
Larsson, 2015	0.014	0.01	44.6%	1.01 [0.99, 1.03]
Wilhelmsen, 2001	−0.008	0.016	30.4%	0.99 [0.96, 1.02]
**Subtotal (95% CI)**			**100.0%**	**1.00 [0.97, 1.02]**
Heterogeneity: Tau² = 0.00; Chi² = 4.16, df = 2 (P = 0.12); I² = 52%				
Test for overall effect: Z = 0.24 (P = 0.81)				
**1.2.3 Women**				
Larsson, 2015	−0.016	0.014	100.0%	0.98 [0.96, 1.01]
**Subtotal (95% CI)**			**100.0%**	**0.98 [0.96, 1.01]**
Heterogeneity: Not applicable				
Test for overall effect: Z = 1.14 (P = 0.25)				

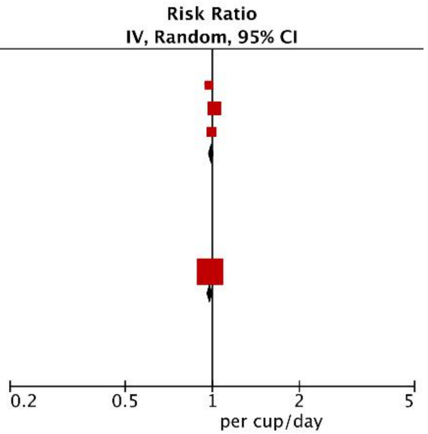

Test for subgroup differences: Chi² = 0.50, df = 1 (P = 0.48), I² = 0%

Supplemental Figure 5. Sex-based analysis for association of per cup/day of coffee intake with AF risk

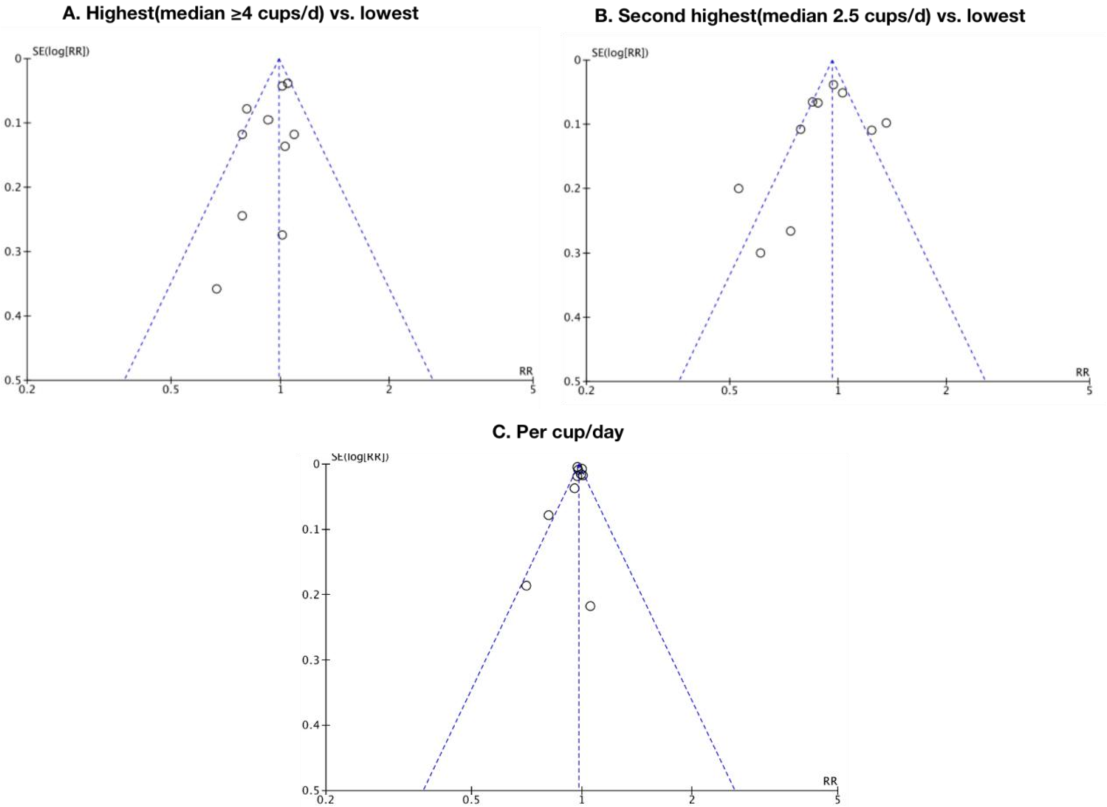

Supplemental Figure 6. The publication bias was assessed using funnel plots

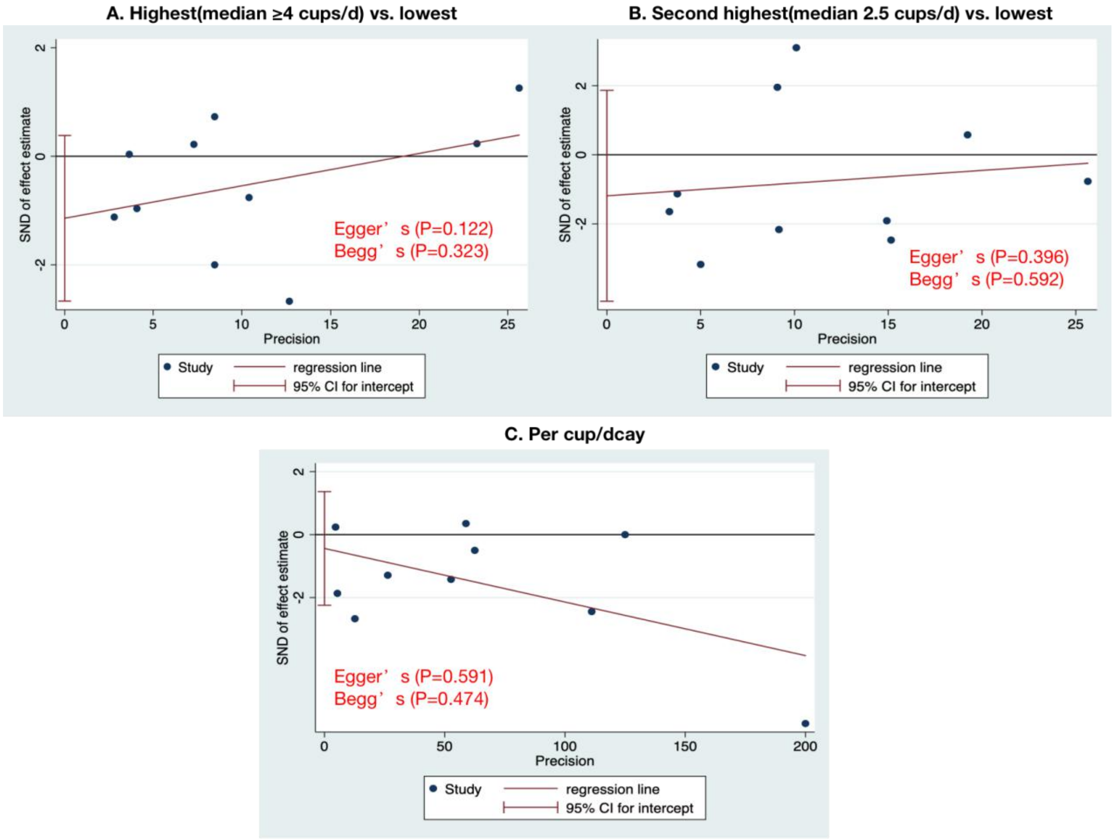

Supplemental Figure 7. The statistical publication bias was assessed using Egger’s and Begg’s tests
